# Mismatch negativity indices and functional outcomes in unipolar and bipolar depression

**DOI:** 10.1038/s41598-020-69776-4

**Published:** 2020-07-30

**Authors:** Sungkean Kim, Ji Hyun Baek, Se-hoon Shim, Young Joon Kwon, Hwa Young Lee, Jae Hyun Yoo, Ji Sun Kim

**Affiliations:** 1 J. Crayton Pruitt Family Department of Biomedical Engineering, University of Florida, Gainesville, FL 32611 USA; 2 Department of Psychiatry, Samsung Medical Center, Seoul, Republic of Korea; 3 Department of Psychiatry, College of Medicine, Soonchunhyang University Cheonan Hospital, 31 Suncheonhyang 6-gil, Dongnam-gu, Cheonan, 31151 Republic of Korea; 4 Department of Psychiatry, Seoul St. Mary’s Hospital, College of Medicine, The Catholic University of Korea, Seoul, Republic of Korea

**Keywords:** Neuroscience, Biomarkers, Diseases

## Abstract

The aim of the study was to explore the association between functional outcomes and mismatch negativity (MMN) activity in participants with mood disorders. The study participants were 27 subjects with major depressive disorder (MDD), 29 subjects with bipolar disorder (BD), and 33 healthy controls who performed a passive auditory oddball paradigm while electroencephalography (EEG) was recorded. Peak amplitudes and source activity of the MMN were compared across groups. Mood and anxiety symptoms were evaluated. The functional levels were the lowest in the BD group, followed by the MDD and healthy control groups. The subjects with BD had significantly lower MMN amplitudes at the frontal and frontocentral electrodes than the healthy controls. The source activity of the MMN from the left anterior cingulate cortex, inferior frontal gyrus, and middle frontal gyrus was significantly increased in the BD group compared to the MDD group. Significant correlations were detected between the functional outcomes and MMN amplitudes at frontal and frontocentral sites. The functional outcome was significantly correlated with left frontal regions. In conclusion, MMN activity appears to be a promising candidate as an evaluation tool for functional outcomes in mood disorders.

## Introduction

Bipolar disorder (BD) and major depressive disorder (MDD) are chronic, severe and recurrent mood disorders with substantial disease burden^1^. As these two psychiatric conditions both involve severe depressive symptoms, misdiagnosis of BD as unipolar depression is a serious clinical problem^2^. Previous studies suggested that C-reactive protein level^3^ and heart rate variability^4^ are candidate biomarkers for discriminating between bipolar depression and unipolar depression. In addition, electroencephalography (EEG) cordance and coherence values may potentially discriminate unipolar from bipolar depression^5^. Although it is important to find biomarkers to enable the differential diagnosis between unipolar and bipolar depression, relatively few studies have addressed this. As such, promising biomarkers to differentiate between the two conditions are needed.

Many patients with BD or MDD usually experience serious functional decline^6^. Depressive symptoms have been shown to be associated with functional role impairments in multiple domains, such as duties at work or school, responsibilities at home, and relationships with family and friends^7^. A previous study found that only 38% patients with major mood disorder achieved functional recovery within 2 years^8^. The psychosocial disability resulting from depression is extensive, and encompasses multiple domains, including work and social interactions, independent living in the community, family adjustment, mortality, and quality of life^6^. However, it remains unclear why functional decline is so pervasive in mood disorders. In addition, very few studies have assessed the specific biomarkers related to functional decline.

Psychosocial functioning is defined as a person’s capability to conduct daily life tasks and to engage in relationships with other people in ways that meet the demands of the community and individual lives that give rise to it^9^. Impairment in social cognition may affect patients’ everyday psychosocial functioning^10^. Social cognition reflects the aspect of cognition dedicated to social information processing for adaptive functioning^11^. More specifically, it represents a sophisticated set of higher-order neuropsychological domains that enable adaptive behaviours in response to others^12^. A previous study revealed that social cognition might be impaired in patients with depression^13^. Significant dysfunctions in theory of mind have been found in BD, in both remitted and sub-syndromal patients, with a greater impairment during acute phases^12,14^. Social cognitive deficits have been identified in both acutely depressed^15^ and remitted patients with MDD^16^.

Mismatch negativity (MMN) is an event-related potential component responding to a sequence of relatively standard stimuli interrupted by the infrequent presentation of deviant stimuli^17^. MMN represents preattentive auditory processing^18^. Näätänen et al. comprehensively reviewed studies which closely correlated MMN with cognitive status^19^. MMN amplitude reduction is considered to be associated with cognitive function in the various domains of cognition in patients with psychosis^7^. In particular, MMN is known to be associated with social cognition and functional outcomes^20^. Previous studies have reported that greater MMN activity correlates with better productivity in the workplace and independent living, and with better social perception in patients with schizophrenia^20^. In addition, functional outcomes were the most powerful predictors of MMN in patients with schizophrenia^21^. In light of MMN reflecting glutamatergic function^22^, MMN reduction may reflect pathological dysfunction in the N-methyl-D-aspartate (NMDA) receptor system^23^.

In this regard, MMN may be a promising biomarker associated with functional decline in mood disorders. MMN and functionality measures are correlated in healthy controls^24^, demonstrating that MMN is independently related to social functioning^24^. This also suggests that an attenuated MMN amplitude to duration deviants may therefore not be specific to schizophrenia, but rather to deficits in cognitive function^25^. However, the association between MMN and functional levels in subjects with mood disorders has yet to be further examined. Patients with BD and MDD experience decreased neurological and social cognitive functioning during illness periods. Given the importance of functional outcomes in BD and MDD, studies to evaluate the relationship between MMN and functional outcomes should be conducted. However, no studies have explored the correlations between MMN and functionality in both psychiatric conditions.

In general, MMN is generated in the primary auditory cortex and in adjacent areas of the superior temporal lobe^26,27^. The prefrontal areas, including the anterior cingulate cortex, inferior frontal gyrus, and middle frontal gyrus, are also considered MMN generators^7,27–30^. The prefrontal generators have been associated with the involuntary switching of attention towards changes in the auditory environment^31^. In particular, the prefrontal generators have been related to a cognitive role or comparator-based mechanism of MMN^32–34^. Additionally, previous studies have demonstrated the roles of the prefrontal cortex in distinguishing between unipolar and bipolar depression^35^. Moreover, the prefrontal cortex may also play a critical role in the functional outcomes of psychiatric diseases^36,37^. Therefore, it is meaningful to investigate MMN activity in the prefrontal regions and the correlation between MMN activity and functional outcomes in BD and MDD patients.

The aim of the study was to assess the association between MMN and functional outcomes in mood disordered and healthy populations. We hypothesized that the amplitude of MMN, reflecting neuro-social cognition, would differ in patients with BD and MDD and healthy populations. Additionally, the functionality measures would be correlate with the amplitude of MMN in both mood disordered and healthy populations. Considering that functional decline is more severe in patients with BD than in those with MDD^38^, we also hypothesized that abnormalities in MMN would be more prominent in patients with BD than in those with MDD. Moreover, previous studies have suggested that age of onset and illness duration may affect functional outcomes in mood disorders^39,40^. Considering previous studies that showed no MMN change in the first episode of affective disorders^41,42^, we also hypothesized that the age of onset and illness duration might correlate the MMN activity as well as functional outcome measures. Finally, we explored the regional activity of the brain through a source activity analysis of the MMN. To support our hypothesis, we verified that brain regions known to be related to functional outcomes were activated in conjunction with changes in MMN.

## Results

### Participants

Table 1 represents the baseline demographic and clinical characteristics in patients with MDD and BD and healthy controls. There were no significant differences in the groups according to age or sex. The healthy control group had significantly more education years than the patient groups (*p* < 0.001). The MDD and BD groups showed no significant differences in terms of years of education. Patients with BD were significantly younger at disease onset (*p* = 0.016) and had longer durations of illness (*p* < 0.001) than the patients with MDD.

**Table 1:** Demographic and clinical characteristics of all study participants.

	MDD^a^ (N = 27)	BD^b^ (N = 29)	Healthy controls^c^ (N = 33)	*P*	Post-hoc (Bonferroni)
Age (years)	34.67 ± 17.44	33.86 ± 14.71	29.85 ± 5.80	0.313	
Sex				0.849	
Male	11 (40.7)	14 (48.3)	15 (45.5)		
Female	16 (59.3)	15 (51.7)	18 (54.5)		
Education (years)	10.48 ± 3.17	11.45 ± 2.60	15.82 ± 1.90	< 0.001	a < c, b < c
Onset age (years)	32.67 ± 16.26	23.66 ± 10.29		0.016	
Duration of illness (months)	26.63 ± 38.70	118.72 ± 123.25		< 0.001	
STAI state	65.33 ± 9.03	59.34 ± 10.69	30.91 ± 8.79	< 0.001	a > c, b > c
STAI trait	64.96 ± 6.33	59.38 ± 11.52	33.85 ± 8.35	< 0.001	a > c, b > c
BDI	55.93 ± 7.05	53.48 ± 11.98	21.33 ± 0.60	< 0.001	a > c, b > c
MDQ	0.41 ± 0.75	8.72 ± 1.73	0.06 ± 0.24	< 0.001	a < b, c < b
GAF	88.89 ± 7.76	83.28 ± 8.27	96.21 ± 4.34	< 0.001	a > b, a < c, b < c
SAS	35.63 ± 8.19	43.14 ± 9.99	25.73 ± 4.79	< 0.001	a < b, a > c, b > c
SFQ	14.11 ± 3.93	23.03 ± 4.14	8.45 ± 0.62	< 0.001	a < b, a > c, b > c

*MDD* major depressive disorder, *BD* bipolar disorder, *STAI* State-Trait Anxiety Inventory, *BDI* Beck Depression Inventory, *MDQ* Mood Disorder Questionnaire, *GAF* Global Assessment of Functioning, *SAS*: Social Adjustment Scale, *SFQ*: Social Functioning Questionnaire.

### Clinical characteristics

Results revealed no significant differences in the State-Trait Anxiety Inventory (STAI) state, STAI trait, or Beck Depression Inventory (BDI) between patients with MDD and patients with BD. Patients with BD showed significantly higher Mood Disorder Questionnaire (MDQ) scores than patients with MDD (*p* < 0.001). In addition, patients with BD showed significantly lower Global Assessment of Functioning (GAF) scores than patients with MDD (*p* = 0.009). Patients with BD showed significantly higher scores on the Korean version of the Social Adjustment Scale (K-SAS) (*p* = 0.002) and Social Functioning Questionnaire (SFQ) (*p* < 0.001) than did patients with MDD. These results indicate that patients with MDD had better functional outcomes than patients with BD.

### Mismatch negativity

**Figure 1:**
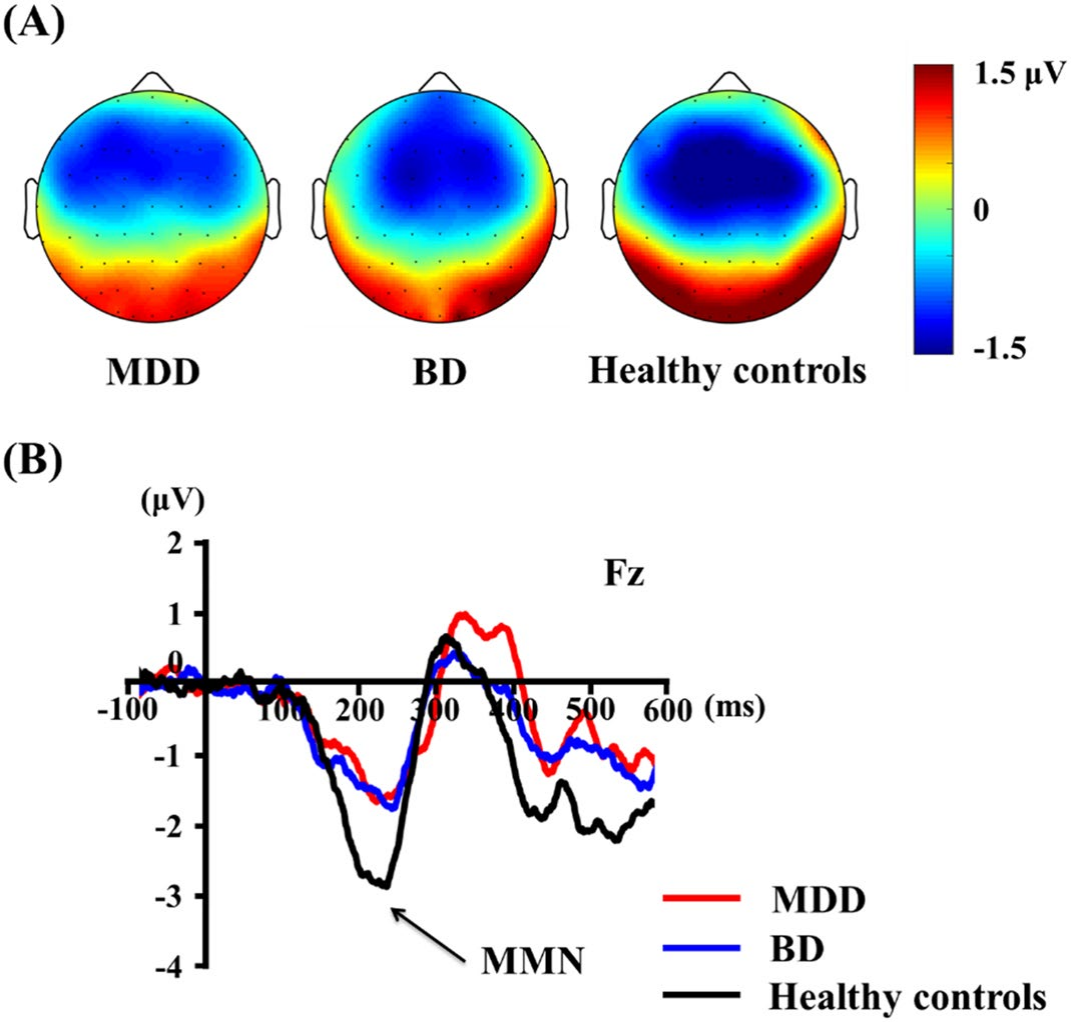
(**A**) Topographic maps of mismatch negativity (MMN), and (**B**) MMN waveforms at the Fz electrode site in patients with major depressive disorder (MDD) and bipolar disorder (BD) and healthy controls.

The grand-averaged MMN waveforms and topographical maps for each group are shown in Fig. 1. A repeated-measures ANOVA assessed MMN amplitudes revealed no significant electrode main effect or group-by-electrode interaction. However, there was a significant main effect of group with middle electrodes (*F* = 4.367 df = 2, *p* = 0.016). Post-hoc tests revealed that the patients with BD showed significantly lower MMN amplitudes than the healthy controls at the middle electrodes (Fz and FCz). A comparison of the peak amplitude of the MMN among the study participants is shown in Table 2.

**Table 2:** Comparison of the mismatch negativity peak amplitudes (µV) among participants.

Site	MDD^a^ (N = 27)	BD^b^ (N = 29)	Healthy controls^c^ (N = 33)	*F*	*P*	Post-hoc (Bonferroni)
Left				2.762	0.069	
F3	− 3.21 ± 1.51	− 2.83 ± 1.27	− 3.63 ± 1.15			
F4	− 3.29 ± 1.61	− 3.23 ± 1.77	− 3.41 ± 2.21			
Middle				4.367	**0.016**	b > c
Fz	− 3.25 ± 1.66	− 2.72 ± 0.97	− 3.81 ± 1.57			
FCz	− 3.25 ± 1.76	− 2.73 ± 1.31	− 3.76 ± 1.58			
Right				0.321	0.726	
F4	− 3.29 ± 1.61	− 3.23 ± 1.77	− 3.41 ± 2.21			
FC4	− 3.04 ± 1.69	− 2.87 ± 1.54	− 3.41 ± 1.57			

Significant value is indicated in bold (p < 0.05)

*MDD* major depressive disorder, *BD* bipolar disorder.

### Source analysis

A repeated-measures ANOVA on MMN source activities revealed no significant region main effect or group-by-region interaction. However, there was a significant main effect of group with left regions (*F* = 3.925, df = 2, *p* = 0.023). Post-hoc tests revealed that the patients with BD showed significantly stronger activation compared to the MDD patients in the left regions (ACC, IFG, and MFG). Detailed information on the source activities of the MMN among the three groups is provided in Table 3.

**Table 3:** Brain regions for source activities of mismatch negativity among participants.

Region of interest	MDD^a^ (N = 27)	BD^b^ (N = 29)	Healthy controls^c^ (N = 33)	*F*	*P*	Post-hoc (Bonferroni)
Left				3.925	**0.023**	a < b
Anterior cingulate cortex	2.05 ± 1.74	7.14 ± 7.02	7.23 ± 7.85			
Inferior frontal gyrus	3.56 ± 3.12	8.21 ± 7.36	7.93 ± 7.35			
Middle frontal gyrus	3.07 ± 2.29	6.83 ± 6.11	6.29 ± 5.46			
Right				2.337	0.103	
Anterior cingulate cortex	2.41 ± 2.01	7.05 ± 6.92	9.56 ± 10.62			
Inferior frontal gyrus	4.16 ± 4.86	8.06 ± 8.17	10.53 ± 8.03			
Middle frontal gyrus	3.83 ± 3.66	6.07 ± 5.73	10.19 ± 9.70			

Significant value is indicated in bold (p < 0.05)

*MDD* major depressive disorder, *BD* bipolar disorder.

### Correlation analysis of MMN activity with clinical symptoms and functions

**Figure 2:**
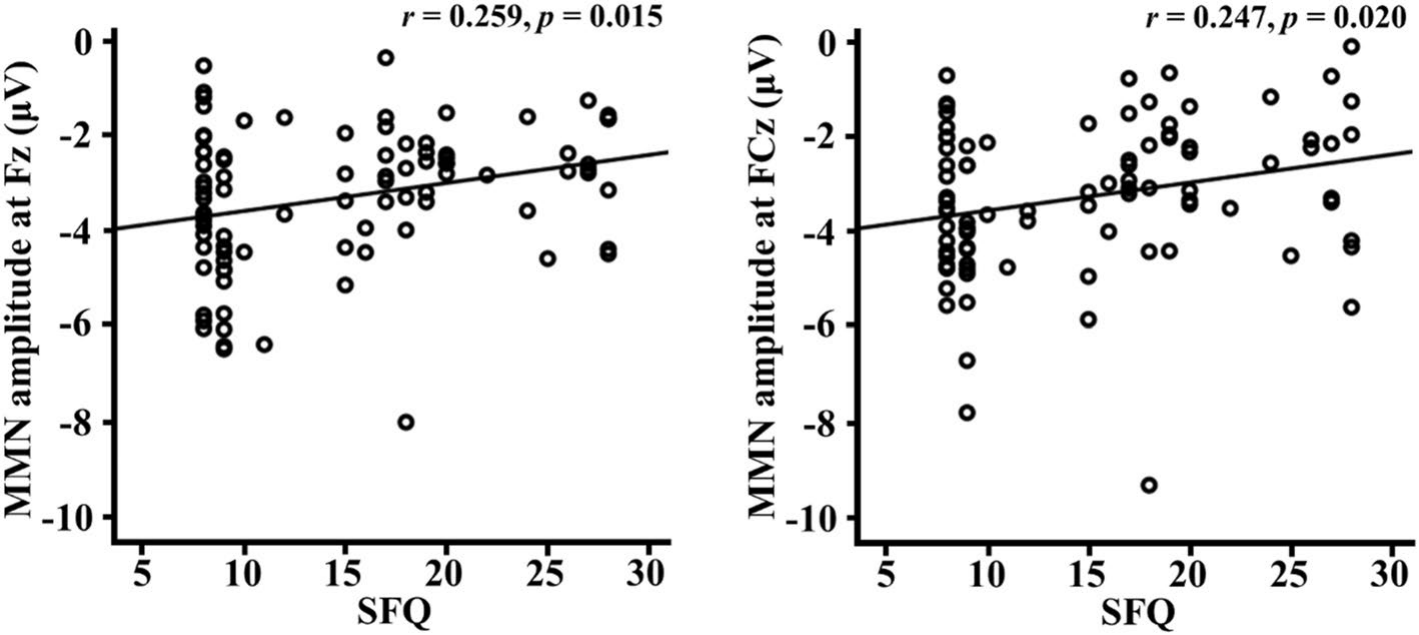
Correlations between MMN amplitudes and psychological measure in all participants. *MMN* mismatch negativity, *SFQ* social functioning questionnaire.

In the correlation analyses, functional outcome measures such as the SAS scores were found to be significantly correlated with age of onset (r = 0.270, *p* = 0.044) and illness duration (r = 0.476, *p* < 0.001). The SFQ scores were also significantly correlated with illness duration (r = 0.476, *p* < 0.001). However, there was no significant correlation between age of onset, illness duration and MMN amplitudes in the patient group. Significant correlations were detected between the psychological measures, including functional outcome measures, and MMN amplitudes at the frontal and frontocentral electrodes in the study population. In the functionality measures, there were significant correlations between the SFQ scores and MMN amplitude at the Fz (r = 0.259, *p* = 0.015) and FCz (r = 0.247, *p* = 0.020) electrodes (Fig. 2). However, there were no significant correlations between GAF scores and MMN amplitudes at the Fz (r = − 0.127, *p* = 0.239) and FCz (r = − 0.132, *p* = 0.219) electrodes. In addition, there were no significant correlations between SAS scores and MMN amplitude at the Fz (r = 0.171, *p* = 0.110) and FCz (r = 0.153, *p* = 0.155) electrodes. Furthermore, there were no significant correlations between the nine subtypes of SAS scores and MMN amplitudes at the Fz and FCz electrodes.

**Figure 3:**
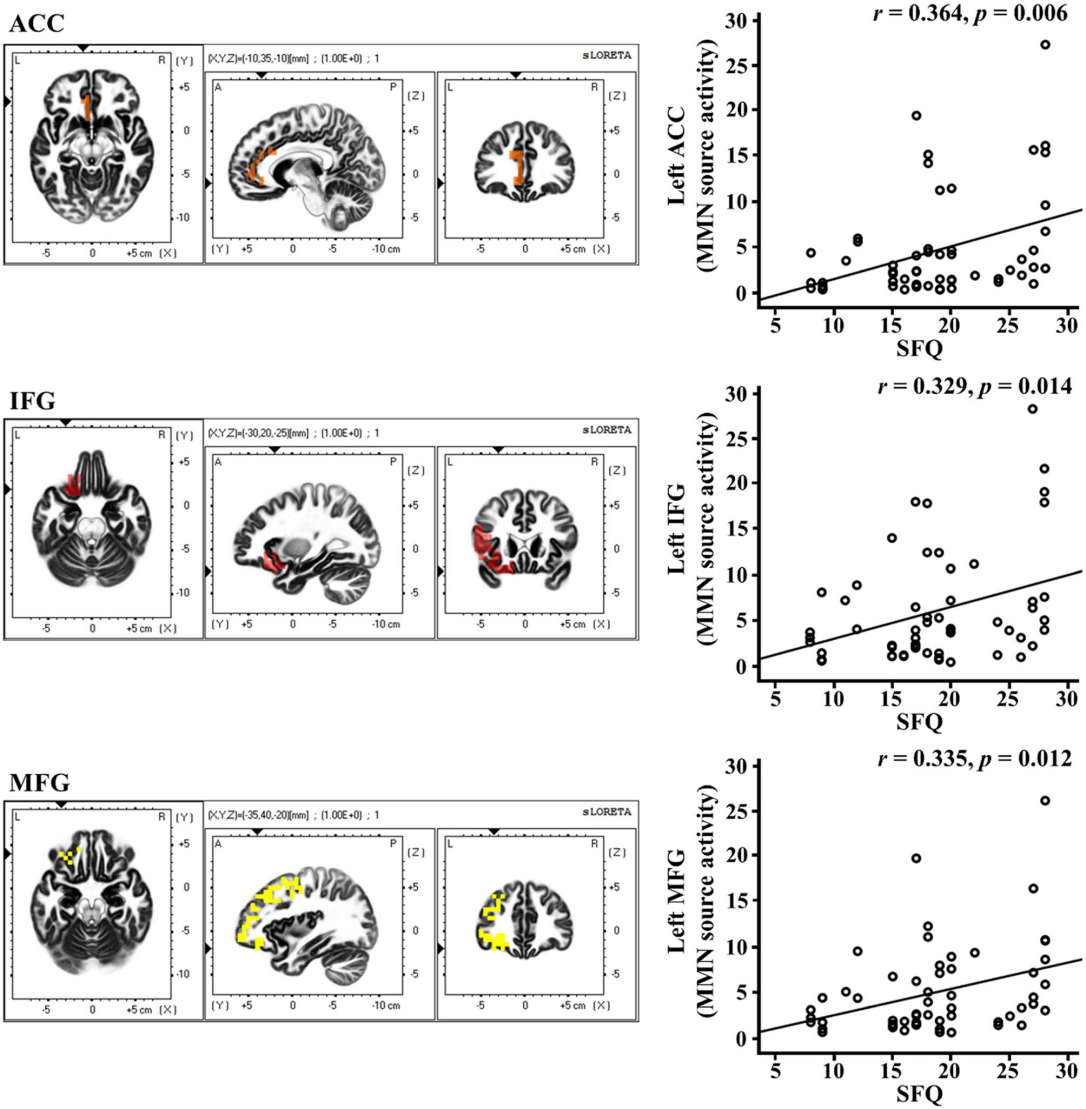
Correlations between MMN source activities and psychological measure in patient groups. *MMN* mismatch negativity, *SFQ* social functioning questionnaire, *ACC* anterior cingulate cortex, *IFG* inferior frontal gyrus, *MFG* middle frontal gyrus.

In the correlation analyses between the source activity of the MMN and psychological measures in the patients, activity in the left inferior frontal gyrus was significantly correlated with the age of onset (r = − 0.310, *p* = 0.021). In addition, activity in the left inferior frontal gyrus and middle frontal gyrus was significantly correlated with the duration of illness (left inferior frontal gyrus: r = 0.384, *p* = 0.004; left middle frontal gyrus: r = 0.480, *p* < 0.001). Moreover, activity in the left ACC, inferior frontal gyrus, and middle frontal gyrus was significantly correlated with the SFQ scores (left ACC: r = 0.364, *p* = 0.006; left inferior frontal gyrus: r = 0.329, *p* = 0.014; left middle frontal gyrus: r = 0.335, *p* = 0.012) (Fig. 3). There were no significant correlations between age of onset and activity in the left ACC and middle frontal gyrus (left ACC: r = − 0.262, *p* = 0.054; left middle frontal gyrus: r = − 0.244, *p* = 0.073). In addition, there was no significant correlation between duration of illness and activity in the left ACC (r = 0.323, *p* = 0.016). Furthermore, there were no significant correlations between GAF scores and activity in the left ACC, inferior frontal gyrus, and middle frontal gyrus (left ACC: r = − 0.146, *p* = 0.287; left inferior frontal gyrus: r = − 0.165, *p* = 0.228; left middle frontal gyrus: r = − 0.184, *p* = 0.179). Finally, there were no significant correlations between SAS scores and activity in the left ACC, inferior frontal gyrus, and middle frontal gyrus (left ACC: r = 0.192, *p* = 0.160; left inferior frontal gyrus: r = 0.195, *p* = 0.154; left middle frontal gyrus: r = 0.229, *p* = 0.093).

## Discussion

This study sought to determine the relationship between MMN activity and functional decline in patients with MDD and BD and healthy populations. To this end, we evaluated the association of MMN amplitude and source activity with functional outcomes in the study participants. As hypothesized, the MMN amplitudes differed between patients and healthy populations. Compared to the previous studies which included patients with psychotic symptoms and various mixed mood states, the present study focused on depressed patients without psychotic symptoms.

As expected, patients with mood disorders showed worse functional outcomes than healthy controls. Those with BD had particularly worse functional outcomes than those with MDD. The duration of illness may affect functional outcomes as well, however, consistently with previous study findings^39^. These results were comparable to those of previous studies whereby impaired MMNs have been shown to be significantly associated with functional impairments, even in healthy populations as well as a range of psychiatric samples^24,43^. Impairments in deviance detection phenomena, even at early stages of processing, may induce significant disturbances in higher-order cognitive functioning and could affect functional outcomes^44^. Moreover, our results also confirmed the previous reports that MMN is independently associated with social functioning, regardless of population characteristics.

This association between MMN peak amplitude and functional outcome was significant in patients with MDD and BD. In particular, the association was mainly detected at frontal and frontocentral electrodes. Similarly, previous neurophysiological studies utilizing MMN as an index of NMDA receptor function displayed impairments in MDD and BD^22,45,46^. Recently, changes in the glutamate system were shown to play a significant role in the evolution of mood disorders^47^. Specifically, abnormal levels of glutamate have been shown in the cerebrospinal fluid, serum, and plasma of patients with MDD and BD^48,49^. Post-mortem studies have reported elevated brain glutamate levels and decreased levels of the glutamatergic NMDA receptor subunits in the frontal cortex of patients with depression^22,50^. Previous meta-analyses revealed that BD was associated with increased glutamine concentrations and decreased MMN, particularly in frontal regions^22^. This finding suggests that the MMN amplitudes in frontal electrodes could serve as a potential marker of the functional decline observed in mood disorders.

Meanwhile, patients with BD showed lower MMN amplitudes at the frontal and frontocentral electrodes compared to healthy controls. There was no difference in MMN amplitude between patients with MDD and healthy controls in the present study. Previous neurophysiological studies utilizing MMN as an index of NMDA receptor function displayed impairments in BD patients^22,45,46^. Our results may reflect impaired pre-attentive information processing in patients with BD, but not in patients with MDD, compared to healthy controls. However, in light of our correlations between MMN and functional outcomes, this may be due to the relatively preserved functional levels of subjects with MDD and healthy population in our study. MMN deficits appear robust in patients with schizophrenia^51^. However, MMN deficits were also discovered in many other severe and persistent mental illnesses such as bipolar, major depressive^51,52^, and obsessive–compulsive disorders^53^. Remarkably, MMN deficits in schizophrenic patients are highly associated with patients’ impairments in daily functioning, level of independence in their community living situation, and functional outcomes^54–56^. Across studies, schizophrenic patients with more severe functional impairments had relatively smaller MMNs than did higher functioning patients^24^. Although patients undergoing first episode psychosis also showed significantly reduced MMN amplitudes compared with controls^57^, the first episode psychosis group also showed significant deficits in attention and verbal learning/memory and strong associations between frontocentral MMN amplitudes and cognitive/psychosocial functioning^57^. Therefore, the results of the present study demonstrate that MMN amplitudes may not be disease-specific marker, but rather markers for functional outcomes related to higher order cognitive and psychosocial functioning. However, previous studies revealed frontal lobe dysfunction in patients with MDD^58,59^ and previous studies showed mixed results with regard to MMN activity in MDD^51,60^. Differences in task designs and in participant characteristics may partly explain these different results. Therefore, changes in MMN amplitudes in MDD patients need to be re-assessed in a future study.

In addition, the age of onset and illness duration were significantly associated with functional outcome measures in the present study. However, the age of onset and illness duration were not related to MMN activity in this study. This may be due to differences within the population of individuals with no psychotic symptoms in this study. In addition, due to the relatively small sample size, there may be no correlation between the above clinical characteristics and MMN amplitudes.

In the source activity of MMN, the left ACC, left IFG, and left MFG showed a stronger activation in BD patients than in MDD patients. The ACC is known to be important in fundamental cognitive processes, including decision-making processes, motivation and problem-solving capacity^61,62^. It contributes, along with other cortical and subcortical neural structures, to the processing of complex emotional behaviours and responses, as well as mood regulation^63^. In addition, disorders of social cognition have long been linked to the structure and function of the ACC^64^. The IFG is involved in the modulation or inhibition of a range of impulsive behaviours^65^. The IFG has also been reported in previous studies on socioeconomic decision making and reappraising social emotions^66,67^. Our results showed that the ACC and IFG activity in BD patients were greater than in MDD patients. Although direct comparisons remain difficult since no previous study has yet evaluated MMN source activity in BD patients, a considerable number of previous studies have been conducted. In the neural model of emotional circuitry in a study by Phillips et al.^68^, patients with BD showed increased activity in the ventral system, including the amygdala, insula, ventral striatum, and ventral ACC^68^, and prefrontal cortex, used to identify the emotional significance of a stimulus, produce affective states, and automatically regulate emotional responses. Phillips et al. explained that over-activation in the ventral system, including the ACC and ventrolateral prefrontal cortex, which is located on the IFG, may underlie the neurobiology of BD^68^. These results were comparable to the results of our source activity analyses. Furthermore, left MFG response to inhibitory errors could predict impulsive behaviours related to substance abuse^69^, and the left MFC is involved in a task that requires executive attention^70^. Since MMN can reflect difficulties in pre-attentive emotional processing in the case of depression^71^, decreased MMN amplitudes and increased activation of the ACC and IFG in patients with BD could be interpreted as reflective of the decreased efficiency of automatically regulated emotional processes and increased demand for more emotionally regulatory efforts and resources. This suggests that patients with BD require more effortful emotional control than patients with MDD, which may result in poorer functional outcomes in BD. In other words, unnecessary excessive activation of the ACC or IFG in pre-attentive processing may prevent proper cognitive responses or problem-solving behaviours in social scenes. Moreover, patients with BD were younger at disease onset than the MDD patients in this study. At the time of the research, BD patients had a longer illness duration than MDD patients did. Interestingly, the left IFG was also inversely correlated with age of onset in the patients in the study. BD symptoms, namely those linked to affective instability, typically emerge in late adolescence and early adulthood^72^. It may be that attempts to regulate emotions resulted in smaller left IFG activation early on in the course of the illness. If this process is repeated, patients with BD will experience greater left IFG activation later in the course of the illness to compensate for this lack of resources. However, the lack of any source activity information in the early phases of BD in the present study limits any further speculation in this regard.

Functional outcomes are closely associated with cognitive function and cognitive impairments, especially poorer executive function, which is, therefore, an important feature of the illness affecting patients’ occupational and academic outcomes^7,38^. More interestingly, the higher the SFQ scores (poorer function), the higher the activity of the prefrontal cortex (left ACC, left IFG, and left MFG) in our results. Increased activity in the prefrontal region of interest reflected the decreased efficiency of the automatically regulated emotional processes. Thus, poor emotional regulation in BD patients could result in poor functional outcomes. Although direct comparisons between both patient groups within a single study are lacking^38^, people with BD generally appear to have a greater degree of cognitive impairment, especially in the frontal lobe, as related to executive function, than those with MDD^38,73^. As such, it is quite plausible that BD patients are more likely to experience poorer functional outcomes.

Although there remains little research on the changes in MMN in affective disorders, whether MMN is conceptualized as a trait marker or a state marker is a critical question to understand its clinical utility^74^. A previous study found that MMN could be a potential trait marker reflecting the global severity of mood disorders^75^. Additionally, remitted patients with depression demonstrated reduced MMN amplitudes which were not related to depressive symptom severity. However, a reduced MMN amplitude was associated with reduced cognitive function and functional outcomes^76^. Therefore, previous studies suggested that MMN may provide a more robust trait-marker that has predictive utility for cognitive decline in affective disorders^76^. However, other studies revealed no statistically significant MMN changes in patients with affective disorder^41,77^ and the contribution of depressive symptom state on MMN cannot be concluded yet. Further studies to conclude the role of MMN as a biomarker of affective disorder will be needed.

There were a few limitations to this study. First, the relatively small sample size of this study should be considered when interpreting the results. Further studies will be needed to confirm the results with larger samples. Second, the patients with MDD and BD were taking medications at the time of testing. Although neither typical nor atypical antipsychotics appear to affect MMN amplitudes in patients with schizophrenia ^52,78^, the benzodiazepine and antidepressants which are frequently used in patients with mood disorders have been known to affect MMN amplitudes in previous studies^79–81^. Therefore, a further study controlling for these medication effects will be needed. Next, the present study was based on a single cross-sectional test to assess functional outcomes and was thus unable to assess changes in functional status. Lastly, this study enrolled depressed patients without psychotic symptoms, meaning that ours results cannot be generalized to entire populations of individuals with MDD and BD.

Despite these limitations, to the best of our knowledge, this study was the first to explore the association between the MMN amplitude and functional outcomes in patients with MDD and BD without psychotic symptoms, as well as the correlates between MMN and functional outcomes in these two psychiatric conditions. These results point to the possibility of MMN as an independent marker of social functioning. Detecting an electrophysiological marker of functional outcomes may support the efforts of clinicians to provide proper treatment for patients who are experiencing depressive symptoms for the first time.

## Methods

### Participants

Participants were recruited from the Psychiatry Department of Soonchunhyang University Cheonan Hospital in Korea. The patients with MDD and BD were diagnosed according to the Structured Clinical Interview for Diagnostic and Statistical Manual of Mental Disorders, 4th edition (DSM-IV) Axis I Psychiatric Disorders^82^. The study was performed on 27 patients with MDD (11 men and 16 women) of a mean age of 34.67 ± 17.44-years-old, and 29 patients with BD (14 men and 15 women) of a mean age of 33.86 ± 14.71-years-old. Among the 29 patients with BD, 11 patients were diagnosed with BD type I and 18 patients with BD type II. Participants with any history of neurological or other severe medical disease were excluded from the study in the initial screening interviews. None of the patients had mental retardation or suffered from alcohol abuse, were undergoing electroconvulsive therapy, or had any head injuries. Thirty-three healthy controls (15 men and 18 women) of a mean age of 29.85 ± 5.80-years-old were recruited through posters displayed in the hospital and advertisements in local newspapers. An initial screening interview was conducted by a board-certified psychiatrist to exclude any subjects with identifiable psychiatric disorders or histories of head injuries or neurological disorders. Each participant had a normal hearing ability, confirmed by the 512-Hz tuning fork test^83^ and all were identified as being right-handed. Nineteen of the patients with MDD were taking medications, such as selective serotonin reuptake inhibitors (fluoxetine, escitalopram, and sertraline) or a serotonin and norepinephrine reuptake inhibitor (duloxetine), or others (mirtazapine). Seventeen of the patients with bipolar disorder were taking mood-stabilizing agents (lithium, valproate, and lamotrigine) with or without atypical antipsychotics (risperidone, quetiapine, aripiprazole, and olanzapine). This study was approved by the Institutional Review Board and Ethics Committee of Soonchunhyang University Cheonan Hospital and all experimental protocols were approved by the committee (2018-10-032). The study was performed in accordance with the approved guidelines. Informed consent was obtained from all study participants.

### Assessment

Depressive and anxiety symptoms were evaluated using the Beck Depression Inventory (BDI)^84^ and the State-Trait Anxiety Inventory (STAI)^85^. The STAI is a commonly used measure of trait and state anxiety and consists of a state anxiety inventory (SAI) and trait anxiety inventory (TAI), comprised of 20 items each^85^. The Korean version of the Mood Disorder Questionnaire (K-MDQ) was used to assess bipolarity. The validity of the K-MDQ, a screening instrument for bipolar disorder, has been tested by Korean researchers, who revealed its high Cronbach's alpha (0.88)^86^. A total K-MDQ score of at least 7 (excluding further two questions) was chosen as the optimal cutoff because it showed good sensitivity (0.75) and specificity (0.69)^86^.

Functional outcomes were measured using the Global Assessment of Functioning (GAF) scale, the Korean version of the Social Adjustment Scale (K-SAS) and the Social Functioning Questionnaire (SFQ). The GAF scale evaluates a subject’s functioning in everyday life^87^. Scores on this scale range from 0 to 100. It subjectively measures social, occupational, and psychological functioning in adults. The Social Adjustment Scale was originally developed by Weissman et al.^88^. The validity and reliability of the Korean version of SAS were confirmed by Kim et al.^89^. It contains 70 questions in total, distributed across nine subtypes (instrumental role, chores, finances, family relationships, social leisure, friend relationships, romantic involvement, sexual adjustment, and personal well-being), and provides a global judgment of the patient’s social adjustment over the past two months. A higher score indicates worse performance. SFQ is an eight-item self-reported scale developed for the quick assessment of perceived social functioning^90^. Scores range from 0 to 24 points, with higher scores indicating worse performance.

### Data acquisition and analysis

EEG data were collected in a sound-attenuated EEG room while each participant performed a passive auditory oddball paradigm. EEG signals were recorded using a NeuroScan SynAmps2 amplifier (Compumedics USA, Charlotte, NC, USA) with 64 Ag–AgCl electrodes mounted on a Quik Cap, using an extended 10–20 placement scheme. The ground electrode was placed on the forehead and the physically linked reference electrode was attached to both mastoids. Vertical electrooculogram (EOG) channels were recorded above and below the left eye. Horizontal EOG channels were acquired at the outer canthus of each eye. The impedance was maintained below 5 kΩ. EEG data were acquired with a band pass filter with cutoff frequencies of 0.1 and 100 Hz at a sampling rate of 1,000 Hz. The procedure for the EEG acquisition followed that of our previous study^91^.

Recorded EEG data were preprocessed using CURRY 8 (Compumedics USA, Charlotte, NC, USA). EEG data were re-referenced to an average reference. Gross artifacts were rejected through visual inspection of an experienced person without any prior information regarding the origin of the data. Artifacts regarding eye blinks or eye movements were removed by a mathematical procedure implemented in the preprocessing software^92^. The data were filtered using a 0.1–30 Hz band-pass filter and epoched from 100 ms pre-stimulus to 600 ms post-stimulus. The epochs were subtracted from the average value of the pre-stimulus interval for baseline correction. If any remaining epochs contained significant physiological artifacts (amplitude exceeding ± 75 μV) in any of the 62 electrode sites, they were excluded from further analysis. Only artifact-free epochs were averaged across the trials and participants for the ERP analysis. The procedure for the preprocessing of EEG followed that of our previous study^91^.

Stimulus presentation onset and EEG recordings were synchronized by E-prime (Psychology Software Tools, Pittsburgh, PA, USA). The auditory stimuli consisted of sounds at 85 dB SPL and 1,000 Hz. Subjects were asked to concentrate on a “Where’s Wally?” picture book without paying attention to the sounds. The MMN wave was extracted by subtracting the ERP wave elicited by standard stimuli from that elicited by deviant stimuli for each subject. Deviant tones lasting 100 ms were presented randomly, interspersed with standard tones lasting 50 ms (10% and 90% probabilities, respectively). Auditory stimulation included 750 stimuli with an inter-stimulus interval of 500 ms. These stimuli were delivered through MDR-D777 headphones (Sony, Tokyo, Japan). The MMN peak amplitude was determined as the most negative peak between 130 and 280 ms at six electrode sites (Fz, F3, F4, FCz, FC3, and FC4)^91^.

### Source imaging

Standardized low-resolution brain electromagnetic tomography (sLORETA) was used to compute the cortical distribution of the standardized source current density of MMN activity. sLORETA is a representative source-imaging method for solving the EEG inverse problem^93^, which assumes that the source activation of a voxel is similar to that of the surrounding voxels when calculating a particular solution, and applies an appropriate standardization of current density. The lead field matrix was computed using a realistic head model segmented based on the Montreal Neurological Institute (MNI) 152 standard template, wherein the three-dimensional solution space was restricted only to the cortical grey matter and hippocampus^94^. The solution space was composed of 6,239 voxels with a 5-mm resolution. Anatomical labels, such as the Brodmann areas, were provided using an appropriate transformation from the MNI to Talairach space^95^.

The MMN source image was analysed between 130 and 280 ms after stimulus onset. We focused on the prefrontal regions as ROIs. The regions of interest (ROIs) related to MMN generators were selected based on the previous neuroimaging and ERP source-localization studies and included the prefrontal areas (ACC, IFG, and MFG)^7,27–30^. The MNI coordinates of the voxels, including the ACC, IFG, and MFG, were provided by sLORETA. The MNI coordinates averaged across voxels belonging to each region were as follows: left ACC: − 7.41, 34.22, 8.79; right ACC: 7.65, 34.02, 7.65; left IFG: − 39.89, 22.14, − 0.28; right IFG: 41.36, 22.04, 0.41; left MFG: − 34.01, 25.19, 29.41; right MFG: 35.98, 24.92, 29.37.

### Statistical analysis

A chi-squared test and one-way analysis of variance (ANOVA) were used to examine differences in the demographic variables and psychological scores between the three groups. A repeated-measures ANOVA was performed to assess MMN amplitudes and source activities. At the sensor level, two electrodes at frontal and frontocentral regions as the within-subject variables with three measures (left, middle, and right) were used. The between-subject variable was the group (MDD, BD, and healthy controls). At the source level, three regions (ACC, IFG, and MFG) as the within-subject variables with two measures (left and right) were used. In addition, the three groups were the between-subjects variable. If any significant main effects were found, post-hoc pairwise comparisons using Bonferroni corrections were conducted between the groups to assess the patterns of MMN activity. Since years of education were significantly different in three groups, these were considered as a covariate in the repeated-measures ANOVA.

A partial Pearson’s correlation was conducted between MMN sensors and source activities and psychological measures, including symptom severity and functionality measures, with a 5,000-bootstrap resampling technique to correct for multiple correlations. The bootstrap test is a weaker method than the Bonferroni test for solving the problem of multiple comparisons. However, the robustness and stability of the bootstrap test have been recognized by previous studies^96–98^. Furthermore, the bootstrap test has been widely used in EEG analyses^99,100^. A partial Pearson’s correlation was performed to control for education as a covariate. The significance level was set to *P* < 0.05 (2-tailed). Statistical analyses were performed using SPSS 21 (SPSS, Inc.).
